# Maize Peroxidase ZmPrx25 Modulates Apoplastic ROS Homeostasis and Promotes Seed Germination and Growth Under Osmotic and Drought Stresses

**DOI:** 10.3390/antiox14091067

**Published:** 2025-08-30

**Authors:** Feixue Zhang, Liangjie Niu, Yingxue Li, Xiaoli Zhou, Hui Zhang, Xiaolin Wu, Hui Liu, Wei Wang

**Affiliations:** 1 State Key Laboratory of High-Efficiency Production of Wheat-Maize Double Cropping, College of Life Sciences, Henan Agricultural University, Zhengzhou 450046, China; 2 Dabie Mountain Laboratory, College of Tea and Food Science, Xinyang Normal University, Xinyang 464000, China

**Keywords:** reactive oxygen species, osmotic stress tolerance, maize apoplast, peroxidase

## Abstract

Drought is one of the major abiotic stresses threatening maize production globally. Under drought stress, maize plants produce excessive reactive oxygen species (ROS), leading to oxidative damage. The apoplast, as the site of substance and signal exchange between plant cells and the external environment, is an important location for the production of ROS under drought stress. Elucidating the ROS scavenging mechanisms in the apoplast is crucial for understanding plant stress responses. However, there is still a lack of research on the ROS scavenging enzymes in maize apoplast and their mediated signaling pathways. We verified that maize peroxidase Prx25 (ZmPrx25) is localized in the apoplast, it scan scavenge hydrogen peroxide (H_2_O_2_), and we systematically investigated the responses of the apoplastic *ZmPrx25*-ROS system to osmotic stress. ROS accumulate in the apoplast of maize mesocotyl in response to osmotic stress and transmit the external osmotic stress signals from the apoplast to the inner cellular compartments. The expression of *ZmPrx25* is highly upregulated in the meristematic regions of maize seedlings under osmotic and oxidative stress. Overexpression of *ZmPrx25* in *Arabidopsis* promoted seed germination and plant growth, significantly enhancing tolerance to osmotic and oxidative stress. This study provides a new perspective on the role of *Prx25* in scavenging ROS under drought stress.

## 1. Introduction

With the intensification of global warming, drought has become one of the major abiotic stresses leading to declining grain production [1]. Maize (*Zea mays* L.), an important food crop and a typical C4 plant, often exhibits phenotypes such as plant wilting and a reduced leaf growth rate under drought stress. Particularly, prolonged drought during the flowering and grain-filling stages can lead to production loss or even complete crop failure in maize [2]. Under drought conditions, maize typically enhances its drought resistance by closing stomata and improving its osmotic adjustment capacity [3]. However, the molecular mechanisms underlying these adaptive strategies remain poorly understood. Therefore, a thorough understanding of the molecular mechanisms underlying maize responses to drought provides a theoretical basis for future strategies aimed at mitigating yield losses.

In various stress environments, plants exhibit a common characteristic during the early response stage—the production of ROS [4]. ROS are important stress signaling molecules in plant cells, and their generation and signal transduction mechanisms have always been a hot and challenging research focus at the forefront of plant stress biology [5]. Plant cells can sense external stress and internal metabolic disturbance signals across various subcellular compartments, and in response, they generate ROS. The main sites of ROS production include chloroplasts [6], mitochondria [7], peroxisomes [8], plasma membranes [9], and the apoplast [10]. Apart from the apoplast—where antioxidant capacity is comparatively low—surplus intracellular ROS are eliminated via antioxidant systems. This process serves to maintain redox homeostasis and ward off oxidative damage [10]. Currently, the processes of ROS production and signaling in intracellular organelles such as chloroplasts [11], mitochondria [12], and peroxisomes [13] are well understood [14]. However, the processes of ROS scavenging and signaling in the apoplast are still poorly understood.

The apoplast is a continuous system that consists of cell walls, intercellular spaces, and dead cell lumens (such as vessels), filled with liquid or air, with cell walls as the main structural component [15]. As a dynamic open region, the apoplast functions as a site where substances and signals are exchanged among plant cells, encompassing many physiological processes, mainly involved in the storage and transport of nutrients and water, cell wall synthesis, and cell growth [10]. As one of the most susceptible areas to environmental stress, the importance of the apoplast in the early perception of environmental stress signals is self-evident [10]. A growing body of evidence demonstrates that apoplastic ROS function is a pivotal early signaling molecule, orchestrating plant growth, development, and adaptive responses to both biotic and abiotic stresses [14,16]. Pathogen infection, drought, salt, low-temperature stress, and soil pollution can all affect physiological processes occurring in the apoplast, leading to increased apoplastic pH and ROS accumulation [16,17]. For example, both drought stress and pathogen attack can trigger the rapid production of ROS in the apoplast, which is essential for stomatal closure [18]. Apoplastic ROS can occur independently of stress-induced abscisic acid (ABA) signaling under water stress, but their levels are also regulated by ABA signaling [19].

Apoplastic peroxidase (Prx) belongs to the plant-specific, Class III peroxidase (EC:1.11.1.7), whcih participates in cell elongation, cell wall component synthesis, camalexin synthesis, auxin metabolism, and plays an indispensable role in the process of clearing ROS [20]. Under biotic or abiotic stress, apoplastic Prx activity generally significantly increases [21,22]. Under biotic stress, gene knockout studies in *Arabidopsis* have demonstrated that the presence of a positive feedback mechanism enhances the defense level of apoplastic ROS signaling [23,24,25]. In maize, apoplastic *Prx35* is associated with seed responses to insects and mechanical damage, and the resistance mechanisms in maize against corn borers and cutworms that Prx35 is involved in are independent of each other [26]. Under abiotic stress, Arabidopsis plants overexpressing *AtPrx3* exhibit higher salt tolerance, and wheat plants overexpressing *TaPrx109-B1* enhance drought resistance [27,28]. In maize, it has been observed that the apoplast might be the earliest site for the detection of exogenous ABA treatment in leaves [29]. Under water stress, the levels of ROS in the apoplast of the maize root growth zone are spatially regulated, and this response holds a vital position in the adaptation of roots to osmotic stress [30]. However, the key Prx responsible for ROS scavenging in the maize apoplast remains unclear.

This study aims to elucidate the role of the maize apoplastic peroxidase ZmPrx25 in ROS homeostasis and drought tolerance by analyzing its tissue-specific expression, subcellular localization, in vitro enzymatic activity, and functional contribution under osmotic and drought stress.

## 2. Materials and Methods

### 2.1. Plant Materials and Growth Conditions

Materials used included hybrid Zhengdan958 and inbred line Chang7-2t. Zhengdan958 is currently widely cultivated in China; Chang7-2t was a drought-tolerant mutant line that was produced by us via ^60^Co-γ irradiation of Chang7-2 seeds [31]. Maize seeds were surface-disinfected with 1% NaClO for 15 min, rinsed thoroughly, soaked for 12 h, evenly placed in seedling trays, and incubated in darkness at 28 °C in a plant growth chamber. Half of the etiolated seedlings were subjected to 15% PEG-6000 treatment targeting the mesocotyl, and the other half were used as controls. After stress treatment, the mesocotyl was collected for Prx activity, as well as ROS and H_2_O_2_ levels. Similarly, for gene expression and physiological analysis, etiolated maize seedlings were separated into two groups: one half was exposed to root stress induced by 15% PEG-6000, while the other half served as untreated controls. Tissues were collected for downstream assays. For protein level analysis, seedlings were further divided into three subgroups: (1) one-third received 15% PEG-6000 root stress for 60 min; (2) the remaining two-thirds were pretreated with either 2 mM of NaN_3_ or 0.2 mM of DPI on roots for 15 min, followed by 10% PEG-6000 stress on the mesocotyl for 60 min. The stress treatment apparatus is shown in Figure S1.

*Arabidopsis* lines were Columbia-0 (Col-0) and *ZmPrx25*-overexpressing *Arabidopsis*. Seeds were surface-sterilized first in 70% ethanol for 5 min, then rinsed with sterile water, and finally treated with 1% NaClO for 5 min. The sterilized seeds were sown on MS medium, vernalized in darkness at 4 °C for 48 h, and then transferred to a growth chamber (22 °C, 16 h light/8 h dark). After 7 d, the seedlings were transplanted into soil composed of a 1:1 mixture of vermiculite and nutrient soil. Some plants were normally irrigated for 15 d, followed by 15 d of drought treatment, after which survival rates were recorded. The remaining plants were irrigated for 21 d and then subjected to drought for 10 d to monitor bolting and physiological indices.

### 2.2. Isolation of Apoplastic Fluid

Apoplast isolation was performed using established methods for maize leaves [32,33]. Mesocotyl segments (about 2.5 cm in length) were washed three times with deionized water, immersed in 10% methanol, and vacuum-infiltrated at −45 kPa for 20 min at room temperature. The segments were bundled together using sealing film. These bundles were placed into 15 mL conical centrifuge tubes equipped with plastic mesh at the bottom and centrifuged at a low speed (1000× *g*, 10 min, 4 °C). The liquid collected at the bottom of the tube was considered apoplastic fluid, which was either used immediately or stored at −80 °C for future use.

### 2.3. Light Microscopy and ROS In Vivo Visualization

Mesocotyl sections were stained with iodine or safranin-fast green and observed under a light microscope. ROS detection was completed using a H_2_DCFDA fluorescent probe (Merck D6883, Rahway, NJ, USA) [34]. Maize seedlings were treated with 50 μM of H_2_DCFDA/PBS (pH 7.4) mist for 30 min. After osmotic stress treatment of the mesocotyl with 10% PEG 6000 + 2% agar for varying durations, the mesocotyls were imaged using an IVIS Lumina S5 fluorescence imaging system (excitation 480 nm/emission 520 nm, f/2, exposure time 2 s).

### 2.4. Bioinformatic Analysis of Prx

Prx family gene sequences were retrieved from the NCBI databases, and 30 maize Prx members were identified that are highly homologous to *ZmPrx25*. Conserved motif analysis was performed using the M; MEME suite. Subcellular localization was predicted based on UniProt annotations combined with Plant-mPLoc analysis. The *Cis*-acting elements in the *ZmPrx25* promoter were recognized through the PlantCARE database.

### 2.5. Transcriptome Profiling by RNA-Seq

Following the isolation of total RNA from 100 mg of leaf tissue using a Qiagen kit (Qiagen, Hilden, Germany), mRNA was enriched via poly (A) selection and sequenced on a BGIseq500 platform (BGI, Shenzhen, China). Raw reads were quality-controlled by removing adapters and low-quality sequences with SOAPnuke. Alignment to the maize reference genome (GCF_000005005.2_B73_RefGen_v4) was performed using HISAT2 (version 2.0.5) [35]. DESeq2 (version 1.34.0) was applied to identify DEGs based on a threshold of |log_2_FC| ≥ 1 and a *q*-value ≤ 0.05 [36]. The raw data have been deposited in the EBI-ENA (https://www.ebi.ac.uk/ena/browser/home, access data 29 August 2025) under accession E-MTAB-11413.

### 2.6. Expression Analysis of ZmPrx25 and qRT-PCR Validation

To verify the expression of *ZmPrx25* genes under stress conditions, quantitative reverse transcription PCR (qRT-PCR) was performed, with *ZmUBI* (*GRMZM2G027378*) as the internal standard. The procedures for RNA extraction and qRT-PCR were as described in previous work [37], and the calculation of relative gene expression was performed via the 2^−ΔΔCT^ method [38]. The gene-specific primers were listed in Table S1

### 2.7. Expression and In Vitro Hydrolysis of the Recombinant ZmPrx25 Protein

The coding sequence (CDS) of *ZmPrx25* (GenBank accessions: LOC100192603, Zm00001d028348) was amplified from maize cDNA, with *Bam*HI and *Xho*I restriction enzyme sites introduced during amplification. The recombinant ZmPrx25 protein was expressed in *E. coli* Rosetta (DE3) cells harboring the pET30a (+)−CDS construct. Expression was induced with 0.2 mM or 0.5 mM of isopropyl-β-D-thiogalactopyranoside IPTG and was carried out at 28 °C for 14 h. The cultures were centrifuged (5000× *g*, 5 min, 4 °C) to collect cell pellets. Then, the cultures were washed with PBS, lysed by boiling, and analyzed using SDS-PAGE to verify protein expression. For protein purification, the cell pellets were resuspended in PBS and lysed by sonication. The lysates were centrifuged to collect the supernatants, which were subjected to affinity purification using Ni-NTA agarose. The bound proteins were recovered using an imidazole gradient, and their purities and sizes were assessed by SDS-PAGE. To further verify the recombinant protein, samples were electrophoretically transferred onto PVDF membranes and analyzed by Western blotting with the anti-His antibody. After incubation with a secondary antibody, signals were detected using an appropriate imaging system. Prx activity was determined using the guaiacol assay.

### 2.8. Transient Expression of ZmPrx25

Transient transformation of tobacco was performed according to the methods of [39]. The CDS of *ZmPrx25* was amplified with *Sal*I/*Bam*HI restriction sites from maize cDNA, cloned, and inserted into the pCAMBIA1302 vector. Tobacco plants were grown for 4–5 weeks under a 16 h light/8 h dark photoperiod at 25 °C. The recombinant *Agrobacterium tumefaciens* carrying the pCAMBIA1302-*ZmPrx25* construct was grown in antibiotic-supplemented LB broth at 28 °C with shaking (200 rpm) for 24 h until turbidity was reached. The bacterial culture (1 mL) was added to 50 mL of antibiotic-containing LB medium and grown to an OD_600_ of 0.5–0.6. Cells were harvested by centrifugation and resuspended in infiltration buffer (10 mM of MgCl_2_, 10 mM of MES, 200 μM of acetosyringone) to a final OD_600_ of 1.0. Silwet-L77 was then added at a ratio of 1:1000, and the suspension was incubated at room temperature for 2–3 h. Tobacco leaves were infiltrated on the abaxial side with the bacterial suspension using a syringe, and the infiltration sites were marked. After infiltration, the plants were kept in darkness for 24 h and then transferred to 21 °C for an additional 2 d of growth. Fluorescence was observed using confocal laser scanning microscopy.

Transient transformation of onion epidermis was performed according to the method described by [40]. The CDS of *ZmPrx25* was amplified with *Sal*I/*Bam*HI restriction sites from maize cDNA, cloned, and inserted into the pYFPLT-C1 vector. The inner epidermis of onion bulb scales was peeled under sterile conditions and incubated in the dark on MS solid medium for 24 h. The epidermal tissue was immersed in a suspension of recombinant *A. tumefaciens* carrying the pYFPLT-*ZmPrx25* vector (OD_600_ = 1.0) for 30 min, then transferred to MS medium for co-cultivation at 28 °C under a 16 h light/8 h dark photoperiod for 2 d. Plasmolysis was induced by treatment with a 30% sucrose solution for 10–20 min, and YFP fluorescence was detected using confocal laser scanning microscopy.

### 2.9. Generation of Arabidopsis Lines Overexpressing ZmPrx25

The pCAMBIA1302-*ZmPrx25* vector was introduced into *A. tumefaciens* strain GV3101 and cultured on LB agar plates containing 50 μg/mL of kanamycin and 25 μg/mL of rifampicin at 28 °C for 48 h. A single positive colony was picked and grown in liquid medium to an OD_600_ of 1.0. Bacterial suspension was collected by centrifugation and resuspended in a solution containing 10% sucrose and 0.01% Silwet-77 (OD_600_ = 1.0), then used to transform *Arabidopsis* at the bolting stage via floral dip. After 24 h of dark incubation, the plants were returned to normal growth conditions, and the infection procedure was repeated 2–3 times at 5–7 d intervals. T0 seeds were harvested, and positive transformants were screened by PCR, followed by the identification of high-expression lines using RT-qPCR. Two lines exhibiting the highest expression of *ZmPrx25* were continuously self-crossed, resulting in the establishment of homozygous transgenic lines by the T3 generation for further analysis.

### 2.10. Measurement of Plant Phenotype and Physiological-Biochemical Indices

*Arabidopsis* seeds were surface-sterilized and cultured for 3 d, during which seed germination was monitored. The primary root length of 7 d seedlings was measured. The bolting status of 30 d *Arabidopsis* plants was recorded. Each trait was recorded with at least three biological replicates. The activities of superoxide dismutase (SOD), Prx, catalase (CAT), and the content of malondialdehyde (MDA) in plants were determined using kits from Beijing Solarbio Science & Technology Co., Ltd. (Beijing, China), following the visible spectrophotometry method, with kit numbers BC0170, BC0090, BC0205, and BC0020, respectively. SOD activity was determined based on a xanthine/xanthine oxidase reaction system that generates superoxide anions (O^2−^), which reduce nitroblue tetrazolium (NBT) to form a blue formazan product with a characteristic absorption at 560 nm. SOD inhibits formazan formation by scavenging O^2−^, and its activity is negatively correlated with the absorbance at 560 nm. Prx activity was calculated by detecting the change in absorbance at 470 nm resulting from its catalysis of H_2_O_2_ oxidation of a specific substrate. CAT activity was quantified by monitoring the rate of decrease in the characteristic absorption peak of H_2_O_2_ at 240 nm due to its catalytic decomposition. MDA content was derived based on its condensation with thiobarbituric acid (TBA) under acidic and high-temperature conditions to produce a brownish-red product with maximum absorption at 532 nm, using the absorbance value for calculation. The specific experimental steps were carried out according to the instruction manual.

## 3. Results

### 3.1. Apoplastic ROS Accumulate Under Osmotic Stress

**Figure 1:**
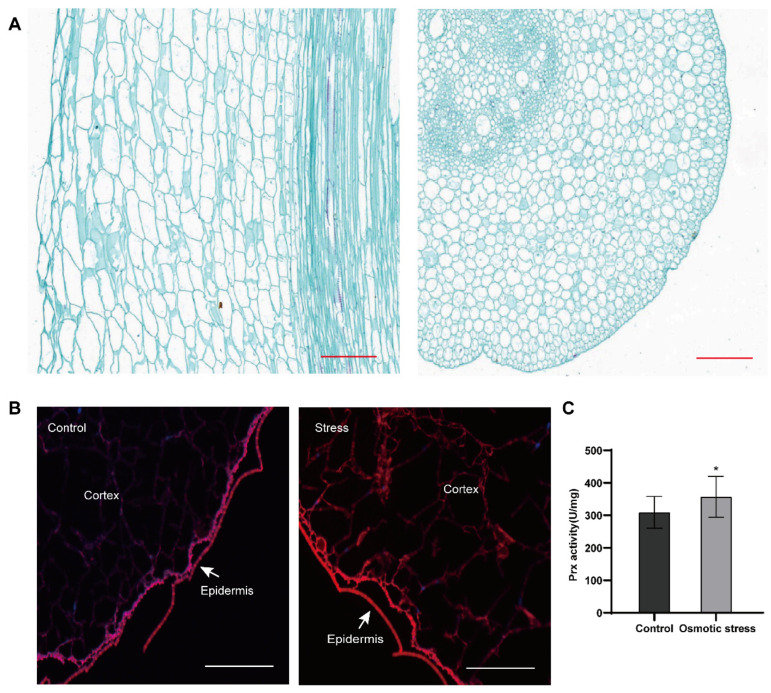
Cellular structure of the maize mesocotyl and ROS fluorescence and detection of apoplastic Prx activity under osmotic stress. (**A**) Cellular structure of the maize mesocotyl. Longitudinal and transverse sections of maize mesocotyl after 132 h of dark treatment, stained with safranin-fast green. Cellulose-rich cell walls appear as green, while lignified or suberized cell walls appear as red. Red lines represent the scale bars, 200 μm. (**B**) Apoplastic ROS fluorescence detection in mesocotyl under 15% PEG 6000 stress. Control, 2% agar; Stress, mesocotyl treated with 2% agar containing 10% PEG 6000 for 30 min. Red fluorescence indicates a significant ROS increase. White lines represent the scale bars, 200 μm. (**C**) Assay of apoplastic Prx activity in maize mesocotyl under osmotic stress. Control, distilled water; Osmotic stress, the mesocotyl was treated with 15% PEG 6000 for 4 h. All experimental data are expressed as mean ± SD. Asterisks indicate statistically significant differences from the control (* *p* < 0.05, analyzed using Student’s *t*-test).

The maize mesocotyl, which connects the coleoptile node and the base of the seed root, plays a critical role in seedling responses to drought, waterlogging, extreme temperatures, and pathogen invasion [41,42,43], inducing stress-related ROS accumulation. Microscopic observations of both longitudinal and cross sections of the mesocotyl revealed that the outermost layer comprised small epidermal cells with thin walls, enclosing the inner cortex and central vascular tissue. The cortical cells were irregular in shape and exhibited large intercellular spaces (Figure 1A,B). These structural features may facilitate the rapid transmission of environmental stress signals via the apoplastic pathway.

To investigate apoplast-specific ROS signaling and transmission under osmotic stress, mesocotyls were treated with 15% PEG 6000. Frozen sections of the mesocotyl stained with dihydroethidium (DHE) and observed under a fluorescence microscope revealed a marked accumulation of ROS in the cell wall and extracellular regions 30 min after osmotic stress treatment, compared to the control, and the ROS signal then progressed inward toward the mesocotyl interior, while intracellular ROS remained at low levels (Figure 1B). This suggests that the observed ROS accumulation is a result of apoplastic sensing of osmotic stress, and that the mesocotyl can sensitively perceive external osmotic stress and transmit the signal inward via apoplastic ROS. Furthermore, Prx activity assays showed a significant increase in apoplastic Prx activity under osmotic stress (Figure 1C), combined with the pattern of ROS accumulation and changes in Prx activity under osmotic stress, suggesting that plants may regulate apoplastic ROS levels by upregulating Prx activity.

### 3.2. Screening for the Major Prx Responsible for Apoplastic ROS Metabolism

**Figure 2:**
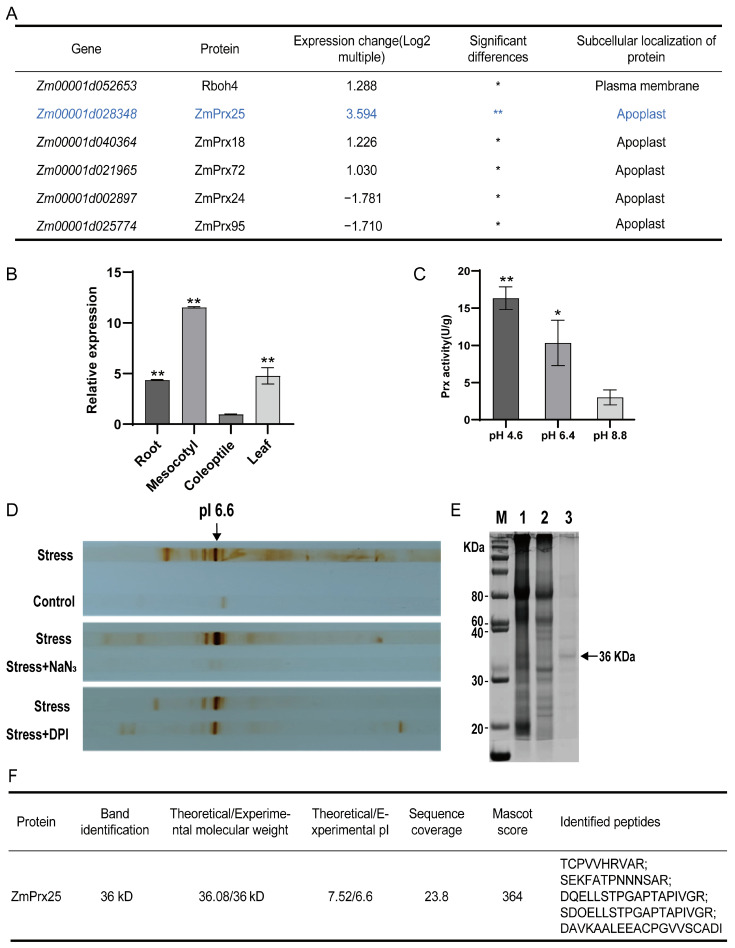
Transcriptomic analysis of *ZmPrx* expression and protein-level validation of ZmPrx25. (**A**) RNA-Seq Analysis of Prx and Rboh Gene Expression in Maize mesocotyl under Osmotic Stress. Log2 > 0, upregulated; Log2 < 0, downregulated. Protein localization based on UniProt annotation. (**B**) The expression of *ZmPrx25* was assessed using qRT-PCR. Chang 7-2t roots grown in darkness for 108 h were treated with 10% PEG 6000 for 90 min, after which *ZmPrx25* expression levels were examined in different organs. All experimental data are expressed as mean ± SD. Asterisks indicate statistically significant differences from the control (* *p* < 0.05, ** *p* < 0.01, analyzed using Student’s *t*-test). (**C**) *Prx* activity in the mesocotyl apoplast at different pH levels. (**D**) Detection of mesocotyl apoplast Prx activity in the presence of specific inhibitors. Prx activity is sensitive to NaN_3_ but not to DPI. Stress, 60 min of Mannitol stress on the mesocotyl; Control, distilled water; Stress + NaN_3_, roots were pretreated with 2 mM of NaN_3_ for 15 min, followed by 10% PEG 6000 stress applied to the mesocotyl for 60 min. Stress + DPI_,_ roots were pretreated with 0.2 mM of DPI for 15 min, followed by 10% PEG 6000 stress applied to the mesocotyl for 60 min, apoplastic proteins from the mesocotyl were separated by IEF. Treatment with stress + DPI indicated that Prx activity was predominantly located at pI 6.6. (**E**) SDS-PAGE analysis of the protein. Lane M, protein marker; Lane 1, proteins extracted from the upper 1 cm segment of the mesocotyl; Lane 2, proteins extracted from the lower 1 cm segment of the mesocotyl; Lane 3, proteins extracted around the pI 6.6 of the gel strips. (**F**) The results of mass spectrometry identification for the 36 kD band.

To determine the major Prx responsible for apoplastic ROS metabolism under osmotic stress, we analyzed the changes in the mesocotyl transcriptomes of Chang7-2t plants exposed to osmotic stress. We identified significant changes in the expression of five Prx genes and *Rboh4*. Among these, the expression level of the *ZmPrx25* gene significantly increased, while the expression of *Rboh4* only increased slightly. The expression levels of the other four Prx genes showed varying degrees of increase or decrease, with the corresponding enzymes located in the apoplast or plasma membrane (Figure 2A). Subsequently, qRT-PCR confirmed that the *ZmPrx25* gene was significantly expressed in the roots, mesocotyls, and leaves of maize seedlings, with the highest expression in the mesocotyl (Figure 2B). the expression trend in the mesocotyl was consistent with the RNA-Seq results mentioned above.

Upon isolating the extracellular fluid from the mesocotyl, Prx activity was measured at different pH values, and it was found that Prx activity was higher under acidic conditions (Figure 2C). Based on the sensitivity of Prx to NaN_3_ and its insensitivity to DPI, roots were pretreated with 2 mM of NaN_3_ and 0.2 mM of DPI, followed by osmotic stress using 10% PEG 6000. Subsequently, apoplastic proteins were separated by isoelectric focusing (IEF), successfully identifying the position of the major Prx induced by osmotic stress on the IEF strip (arrow; Figure 2D). The protein band at pI 6.6 was isolated, and proteins were extracted and analyzed via SDS-PAGE followed by Coomassie blue staining. A band at 36 kDa showed a molecular weight similar to ZmPrx25. The 36 kDa band (Figure 2E,F) was excised for mass spectrometry analysis, which identified ZmPrx25 as the major protein. Analysis of both transcriptomic and protein level demonstrated that ZmPrx25 may be the key enzyme in the apoplast of maize mesocotyl in response to osmotic stress.

### 3.3. Transient Expression and Promoter Analysis of ZmPrx25

**Figure 3:**
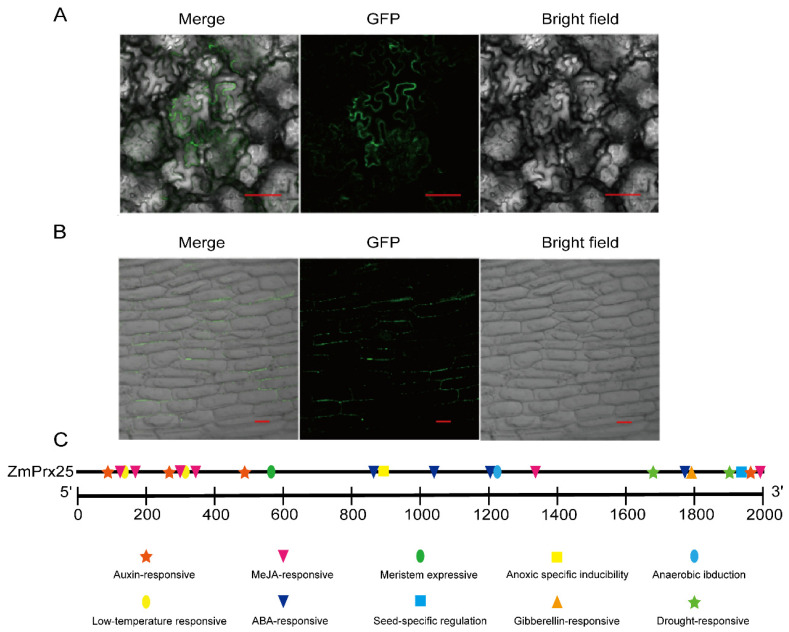
Transient expression and promoter *cis*-element analysis of *ZmPrx25*. (**A**) Transient expression of ZmPrx25 in *N. benthamiana* epidermal cells. (**B**) Transient expression of ZmPrx25 in onion epidermal cells after plasmolysis. Merge, overlay of two kinds of signals; GFP, green fluorescence signal; Bright field, image under light field. Red lines represent the scale bars, 100 μm. (**C**) *Cis*-acting elements in the *ZmPrx25* promoter.

To determine the transient expression of *ZmPrx25*, we constructed pCAMBIA1302-*ZmPrx25* and pYFPLT-*ZmPrx25* vectors and transiently expressed them in *Nicotiana benthamiana* and onion epidermal cells, respectively. Fluorescence observation in *N. benthamiana* indicated that *ZmPrx25* was localized to the plasma membrane or cell wall (Figure 3A). Further plasmolysis experiments in onion epidermal cells confirmed that *ZmPrx25* specifically localized to the cell wall (Figure 3B), which was consistent with the prediction in the UniProt database. Additionally, bioinformatic analysis revealed that the promoter region of *ZmPrx25* contains multiple *cis*-elements that are responsive to drought, low temperatures, and hormones, including ABA, gibberellins (GA), and IAA (Figure 3C), suggesting that this gene may be involved in drought stress response and developmental regulation in maize.

### 3.4. Enzyme Activity of the Recombinant ZmPrx25 Protein

**Figure 4:**
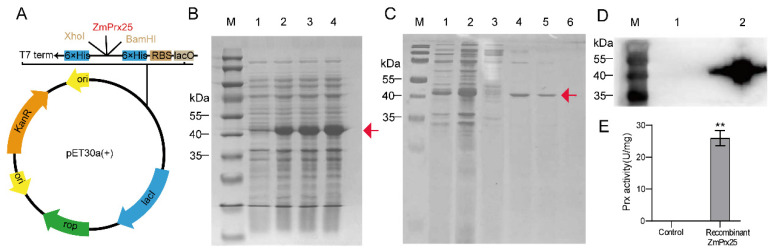
Prokaryotic expression, purification, and enzymatic activity of the recombinant ZmPrx25. (**A**) Establishment of the prokaryotic expression vector pET30a (+)−*ZmPrx25*. (**B**) Expression of the recombinant ZmPrx25 protein. Lane M, protein marker; Lane 1, recombinant ZmPrx25 was not induced; Lane 2, the recombinant ZmPrx25 was induced by 0.2 mM of IPTG for 12 h; Lane 3, induction for 14 h; Lane 4, induction for 16 h. The red arrow indicates the ZmPrx25 protein. (**C**) SDS-PAGE analysis of the recombinant ZmPrx25 purified by Ni-NTA affinity chromatography. After induction with 0.2 mM of IPTG for 12 h, the recombinant ZmPrx25 was purified using different imidazole concentration gradients. The red arrow indicates the ZmPrx25 protein. Lane M, protein marker; Lane 1, PBS buffer; Lane 2, 0 mM of imidazole; Lane 3, 20 mM of imidazole; Lane 4, 50 mM of imidazole; Lane 5, 100 mM of imidazole; Lane 6, 200 mM of imidazole. (**D**) Western blot analysis of the recombinant ZmPrx25. Lane M, protein marker; Lane 1, pET30a (control); Lane 2, the recombinant ZmPrx25 (purified target protein). (**E**) Prx activity assay. All experimental data are expressed as mean ± SD. Asterisks indicate statistically significant differences from the control (** *p* < 0.01, analyzed using Student’s *t*-test).

To determine whether ZmPrx25 can scavenge ROS, the prokaryotic expression vector pET30a (+)−*ZmPrx25* was transformed into *E. coli* competent cells (Figure 4A), which expressed recombinant ZmPrx25. Upon induction with 0.2 mM of IPTG for 12 h, 14 h, and 16 h, a distinct protein band of approximately 41 kDa was observed (Figure 4B), consistent with the predicted molecular weight of the recombinant protein, indicating the successful expression of ZmPrx25. Subsequent purification using imidazole concentrations revealed a single protein band under 100 mM of imidazole elution (Figure 4C). Western blot analysis confirmed that the purified protein was the recombinant ZmPrx25 (Figure 4D). Enzyme activity assays revealed that ZmPrx25 possessed Prx activity, and was capable of efficiently decomposing the substrate H_2_O_2_ (Figure 4E), indicating its enzymatic function in scavenging ROS.

### 3.5. Tissue-Specific Expression Patterns of ZmPrx25 in Maize

**Figure 5:**
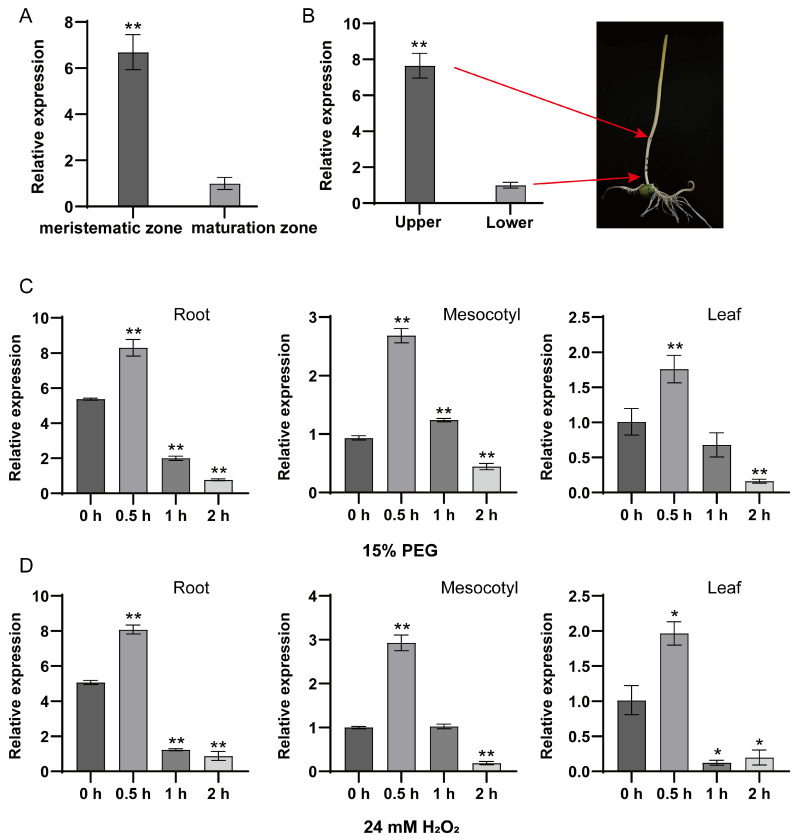
Expression analysis of *ZmPrx25* in different tissues of etiolated maize seedlings and under osmotic and oxidative stress conditions. Different tissues were collected from 108 h etiolated maize seedlings grown in darkness. (**A**) Gene expression of *ZmPrx25* in the meristematic and maturation zones of maize roots. (**B**) Gene expression of *ZmPrx25* in the one cm ends of the mesocotyl. (**C**) Gene expression of *ZmPrx25* in maize roots, mesocotyl, and leaves under 15% PEG 6000 stress. (**D**) Gene expression of *ZmPrx25* in maize roots, mesocotyl, and leaves under 24 mM of H_2_O_2_ stress. All experimental data are expressed as mean ± SD. Asterisks indicate statistically significant differences from the control (* *p* < 0.05, ** *p* < 0.01, analyzed using Student’s *t*-test).

To investigate the function of *ZmPrx25* in maize growth and development, we performed a spatial expression analysis. The results indicated that *ZmPrx25* was significantly expressed in the root meristematic zone (Figure 5A) and at the one cm ends of the mesocotyl (Figure 5B), regions characterized by active cell division. These findings suggest that *ZmPrx25* may play a role in regulating cell proliferation during early developmental stages.

To investigate the role of *ZmPrx25* in stress responses, we treated plants with 15% PEG 6000 and 24 mM of H_2_O_2_. Temporal expression analysis revealed that *ZmPrx25* was rapidly induced by both stress conditions, with transcript levels in roots, mesocotyls, and leaves peaking at 0.5 h. As the duration of stress extended (1–2 h), its expression levels gradually declined (Figure 5C,D). This dynamic expression profile implies a potential functional significance of ZmPrx25 during the initial phase of stress response in maize.

### 3.6. Phenotypic and Drought Tolerance Analysis of ZmPrx25 Overexpression Lines

**Figure 6:**
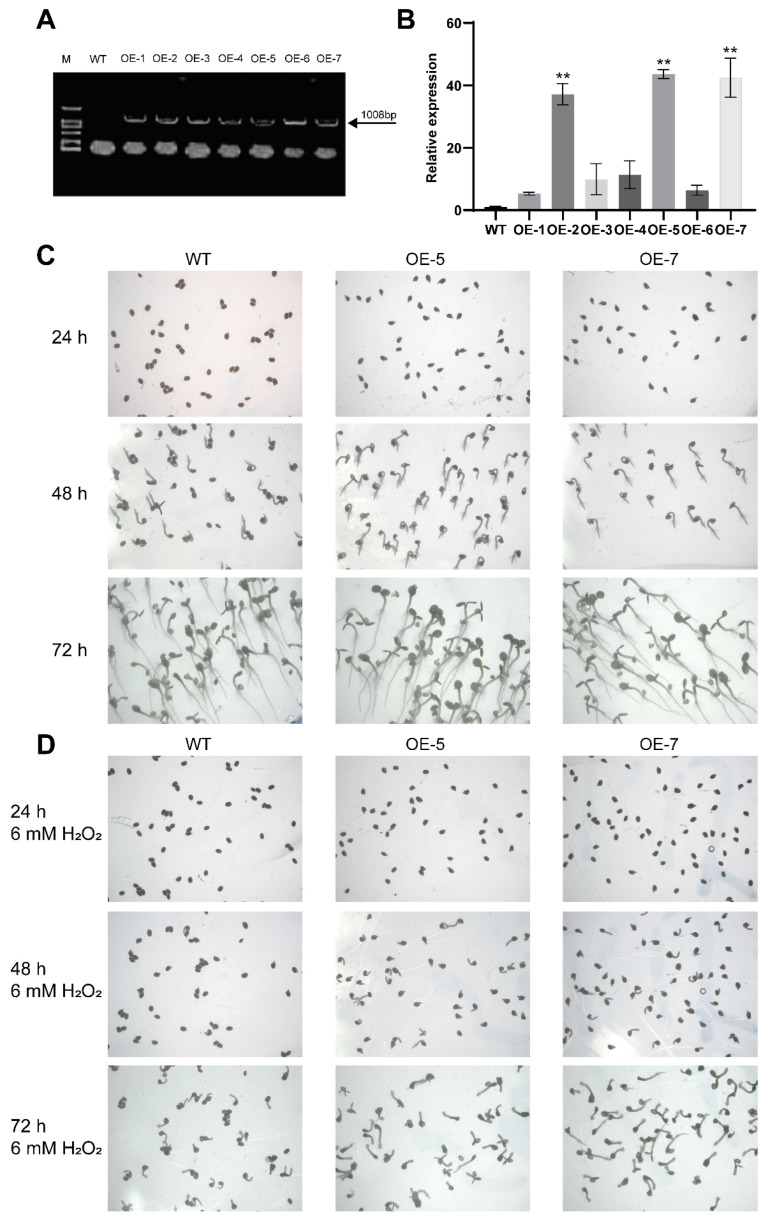
Germination phenotype of *ZmPrx25* overexpressing *Arabidopsis*. (**A**) PCR amplification results of leaf DNA. The arrow points to the location of the target amplicon. Lanes M, WT, and OE-1 to OE-7 correspond to the DNA marker, wild-type, and ZmPrx25-overexpressing lines, respectively. (**B**) RT-qPCR amplification analysis of leaf RNA. Data were obtained from three biological replicates, and significant differences were assessed via Tukey’s test (** *p* < 0.01). (**C**) Seed germination of *Arabidopsis* (1–3 d). (**D**) Seed germination of *Arabidopsis* under oxidative stress conditions (1–3 d).

The construct pCAMBIA1302-*ZmPrx25* vector was successfully introduced into *Arabidopsis* (Figure 6A). Transgenic lines were screened using RT-qPCR, and two lines with high *ZmPrx25* expression, OE-5 and OE-7, were selected for propagation. T3 generation plants were obtained and used for subsequent analyses (Figure 6B).

In the seed germination assay, the germination rates of wild-type (WT) and *ZmPrx25* overexpression (OE-5, OE-7) *Arabidopsis* seeds were assessed after 24 h of cultivation. The results showed that the *ZmPrx25*-OE lines exhibited a germination rate of 98%, whereas the WT seeds displayed only 38% germination at the same time point. Complete germination of WT seeds was observed at 72 h (Figure 6C). To further evaluate the role of *ZmPrx25* in the oxidative stress response, seed germination was examined on MS medium supplemented with various concentrations of H_2_O_2_. The results indicate that *Arabidopsis* overexpressing *ZmPrx25* maintains a high germination rate under oxidative stress conditions (Figure 6D). Notably, under the 6 mM H_2_O_2_ treatment, the germination rate of transgenic lines exceeded 73% within 24 h, while the WT seeds exhibited only 4% germination (Figure S2). These findings indicate that the overexpression of *ZmPrx25* effectively enhances oxidative stress tolerance and significantly promotes seed germination in *Arabidopsis*.

**Figure 7:**
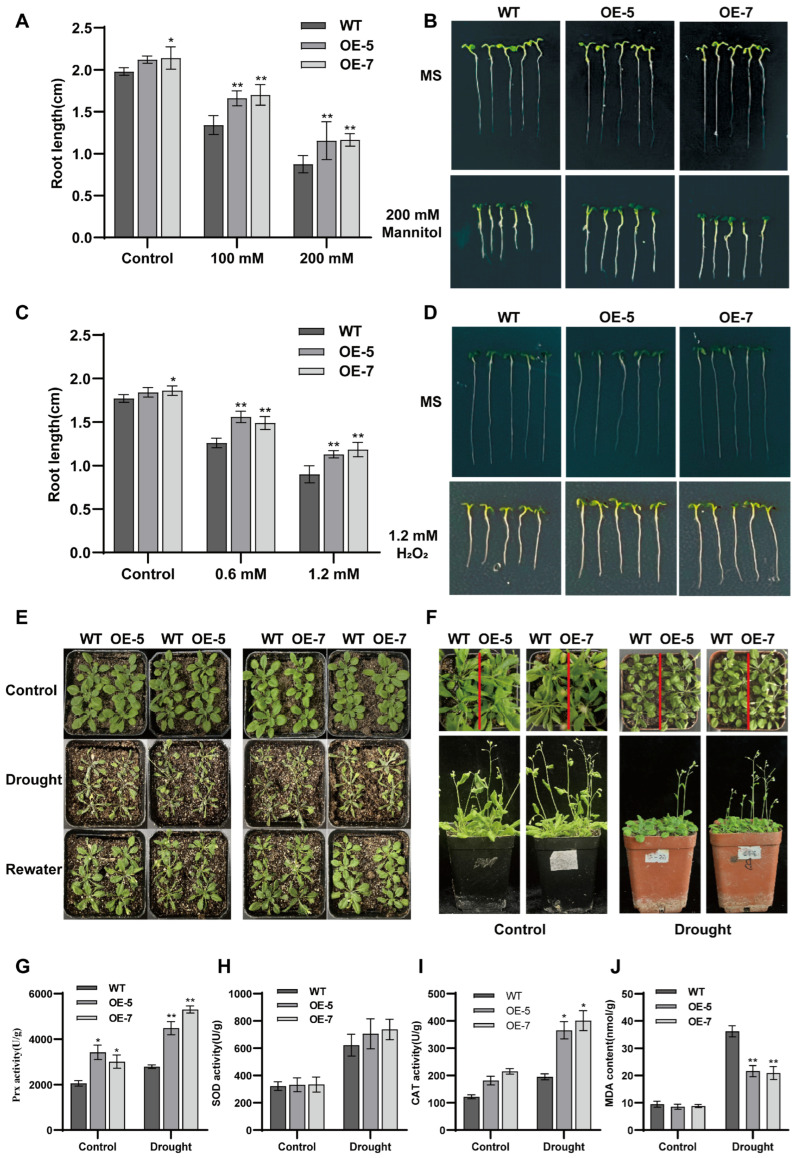
Phenotypic and drought tolerance analysis of transgenic *Arabidopsis* roots under stress. (**A**) Statistical analysis of root length under 100 mM and 200 mM of mannitol stress for 7 d. (**B**) Root phenotype of seedlings grown on 200 mM of mannitol for 7 d. (**C**) Statistical analysis of root length under 0.6 mM and 1.2 mM of H_2_O_2_ stress for 7 d. (**D**) Root phenotype of seedlings grown on 1.2 mM of H_2_O_2_ for 7 d. (**E**) Plant survival of 15 d seedlings under drought stress. Control, normal growth; Drought, water withholding for 15 d; Rewater, water withholding for 15 d and 2 d of re-watering. (**F**) Bolting performance of plants under drought stress. Control, normal growth of 21 d seedlings; Drought, 21 d seedlings after 10 d of water withholding. The red line is used to distinguish WT seedlings from OE seedlings. (**G**–**I**) Activities of antioxidant enzymes (SOD, CAT), and Prx in plants under drought stress. (**J**) Content of MDA in plants under drought stress. All conditions were identical to those of F. All experimental data are expressed as mean ± SD. Asterisks indicate statistically significant differences from the control (* *p* < 0.05, ** *p* < 0.01, analyzed using Student’s *t*-test).

Under both osmotic stress (100 and 200 mM of mannitol) and oxidative stress (0.6 and 1.2 mM o H_2_O_2_), root growth in the *ZmPrx25*-OE Arabidopsis lines was significantly less inhibited compared to the WT plants (Figure 7A–D). Notably, under the 200 mM mannitol treatment, root length in the OE lines was approximately 36–48% greater than that of WT (Figure 7A). These results suggest that the overexpression of *ZmPrx25* enhances tolerance to both osmotic and oxidative stress, thereby promoting root growth under environmental stress conditions.

Under drought treatment, there was no significant difference in survival rates between *ZmPrx25*-OE lines and WT plants after withholding water for 15 d in seedlings (Figure 7E and Figure S2B). However, in 21 d plants subjected to a 10 d drought period, *ZmPrx25*-OE lines exhibited earlier bolting compared to WT (Figure 7F), indicating that *ZmPrx25* may facilitate drought adaptation by accelerating developmental transitions without compromising rosette leaf development. Further physiological analyses revealed that, under drought stress, *ZmPrx25*-OE lines displayed significantly higher Prx and CAT activities, along with lower MDA accumulation, compared to WT plants (Figure 7G–J). These results suggest that *ZmPrx25* enhances drought tolerance by mitigating oxidative damage through increased antioxidant enzyme activity.

Overexpression of *ZmPrx25* enhances stress tolerance in *Arabidopsis* through a dual mechanism: by directly promoting root growth under short-term osmotic and oxidative stress, and by improving plant survival under prolonged drought stress via the activation of enzymatic antioxidant defenses and the regulation of developmental timing.

## 4. Discussion

Plants rapidly generate ROS in multiple cellular compartments as an integral early response to stress [10]. The apoplast, a vital conduit for intercellular signaling, is one of the primary sites where plants perceive and respond to environmental stress [15]. Studies have shown that under stress conditions (e.g., pathogen interaction), the apoplast generated ROS and participated in the early signal transduction process [10]. This study found that the mesocotyl could sensitively detect changes in external osmotic stress and effectively transmit stress signals to internal tissues through apoplastic ROS. Under osmotic stress, the activity of apoplastic Prx was significantly upregulated, combined with the specific accumulation and signaling of ROS in the apoplast, suggesting that plants may regulate the activity of peroxidases like Prx to maintain intracellular ROS homeostasis and prevent oxidative damage [44]. Although the apoplast isolation method used for detecting apoplastic Prx activity in this study meets the needs of basic research, it is difficult to completely exclude cytoplasmic contamination during the isolation process, which may affect the rigor of the experimental results. The existing studies support the role of Prx in apoplastic ROS regulation [44]. Further investigations requiring accurate dissection of apoplastic Prx functions would benefit from improved isolation methodologies to minimize the influence of cytoplasmic contaminants on enzymatic activity measurements.

The production of apoplastic ROS depends on the activation of Prx and Rboh activities [24,25]. Among them, Prx, which is unique to plants, plays crucial roles in various physiological and developmental processes [45], including the regulation of ROS levels, cell wall production and maintenance, lignification, fruit growth, seed germination, hormone metabolism, and protection against various stresses (such as drought and high temperatures). Studies have found that the overexpression of the *GsPrx9* gene enhances plant salt tolerance, promotes antioxidant responses, and reduces ROS accumulation [46]. Furthermore, heterologous expression of *CaPrx2* in *Arabidopsis* resulted in a significant increase in H_2_O_2_ in transgenic plants [47]. Mechanistic validation revealed that sodium azide (NaN_3_) (a Prx inhibitor) effectively suppressed H_2_O_2_ accumulation, whereas diphenyleneiodonium chloride (DPI) (an NADPH oxidase inhibitor) showed no such inhibitory effect, indicating that *CaPrx2* may be involved in the H_2_O_2_ production process [48]. This study confirmed that Prx played a dual function: they mitigated oxidative damage by scavenging excess ROS and participated in ROS-mediated signal transduction processes [49]. This dual function makes Prx a key regulator in maintaining cellular redox balance [50]. Numerous Prx family members have been identified in *Arabidopsis*, rice, and maize, but functional studies are mainly focused on dicots like *Arabidopsis* [51,52], with less research on the function and mechanisms of Prx in important cereal crops like maize.

Prx family proteins are characterized by highly conserved structural domains with a molecular weight of about 28 to 60 kDa [53]. The ZmPrx25 protein identified in this study matched the characteristics of the aforementioned members and showed significant upregulation under osmotic and oxidative stress conditions, similar to the expression patterns of known stress-responsive homologous proteins such as *AtPrx3* [27] and *CrPrx1* [54], suggesting that *ZmPrx25* may play an important role in plant water stress responses. Prx family proteins are typically localized to the cell wall, where they regulate cell wall dynamics by catalyzing the production, scavenging, and oxidation of ROS, proteins, and phenolic compounds [20]. This study confirmed that *ZmPrx25* was localized to the cell wall and is highly expressed in young, dividing tissues. Previous studies have reported that some peroxidases (Prxs) can promote cell elongation by generating hydroxyl radicals (^•^OH) that mediate the oxidative cleavage of cell wall polysaccharides [55]. However, although ZmPrx25 shows high expression in rapidly dividing young tissues, there is currently no experimental evidence indicating whether it regulates cell elongation through a similar “^•^OH-polysaccharide oxidation” mechanism. The direct causal relationship between its high expression and cell elongation phenotypes remains to be further verified.

Functional studies indicate that *ZmPrx25* regulates seed germination through a dual mechanism: on the one hand, this is done by inhibiting the synthesis of ABA and promoting the accumulation of GA to balance hormone metabolism [56]; on the other hand, under oxidative stress induced by H_2_O_2_, overexpressed lines can clear excessive intracellular H_2_O_2_ to maintain ROS homeostasis, thereby accelerating germination and increasing germination rates, consistent with the function of cell wall-localized *AtPrx36* [55]. In root development, the overexpression of *ZmPrx25* catalyzed hydroxyl radicals (^•^OH) to promote cell wall loosening, significantly elongating the primary root of Arabidopsis under normal conditions and maintaining a growth advantage under osmotic/oxidative stress [57]. The overexpression of *ZmPrx25* may catalyze the production of hydroxyl radicals (^•^OH), promoting the relaxation of the cell wall and significantly elongating the primary roots of *Arabidopsis* under normal conditions, while maintaining a growth advantage under osmotic/oxidative stress [57]. This is consistent with findings on *Arabidopsis AtPrx33/34*, where the overexpression of *AtPrx34* resulted in root elongation, whereas *AtPrx33* mutants exhibited root shortening. The analysis of gene expression level of *ZmPrx25* and the protein level of ZmPrx25 in maize were performed under PEG-6000 stress. Considering that PEG does not reproduce the dynamics of soil drought and may yield phenotypes that differ from soil conditions, we employed a soil-drying assay to verify the drought-resistance function of ZmPrx25. Twenty-one-day-old Arabidopsis transgenic plants were subjected to water withholding for 15 days to evaluate drought-stress phenotypes. By integrating both approaches—PEG for rapid gene-expression screening and soil drought for functional confirmation—we provide a more comprehensive assessment of ZmPrx25’s role in drought tolerance. During drought stress, overexpressing lines utilized a “developmental escape” strategy by bolting early to avoid prolonged stress, a process accompanied by increased Prx activity and the synergistic activation of other antioxidant enzymes like CAT, forming a multi-level enzyme-driven antioxidant defense system [58]. However, this study focused solely on *ZmPrx25* and cannot fully rule out potential functional redundancy among other members of the Prx family. Subsequent studies need to conduct more systematic functional analyses of the maize Prx family members.

Under PEG-6000 osmotic stress, transcripts of *ZmPrx25*, *ZmPrx18*, and *ZmPrx72* were significantly upregulated (log_2_FC = 3.594, 1.226 and 1.030, respectively), whereas *ZmPrx24* and *ZmPrx95* were markedly downregulated (Figure 2A). Among the three induced genes, *ZmPrx25* exhibited the strongest induction, providing the rationale for selecting it as the primary candidate for drought/osmotic-stress tolerance in this study. ZmPrx25, ZmPrx18, and ZmPrx72 may exhibit functional redundancy. Future work will employ CRISPR/Cas9-mediated multiplex editing to generate single, double, and triple knock-out lines to dissect their individual and overlapping contributions.

## 5. Conclusions

This study systematically elucidates the central role of the maize peroxidase ZmPrx25 in apoplastic ROS signaling and plant drought/oxidative stress tolerance. In response to osmotic stress, the mesocotyl generates ROS and transmits external osmotic stress signals internally. This process is mediated by the ZmPrx25. This protein maintains ROS homeostasis by scavenging extracellular H_2_O_2_, thereby mitigating oxidative stress and enhancing drought resistance. Overexpression of *ZmPrx25* promotes seed germination and root growth, strengthens the antioxidant system, and induces a drought escape strategy via early bolting. This research provides a theoretical foundation and a valuable gene resource for drought-resistance breeding in crops.

## Data Availability

The original contributions presented in this study are included in the article/Supplementary Material. Further inquiries can be directed to the corresponding author(s).

## Supplementary Materials

The following supporting information can be downloaded at https://www.mdpi.com/article/10.3390/antiox14091067/s1: Figure S1: Mesocotyl osmotic stress apparatus. Figure S2: Seed germination rates of Arabidopsis under oxidative stress and survival rates under drought stress. Table S1: Primers used in this study.

## References

[1] Tang H., Zhang L., Xie X., Wang Y., Wang T., Liu C. (2025). Resilience of maize to environmental stress: Insights into drought and heat tolerance. Int. J. Mol. Sci..

[2] Liu S., Qin F. (2021). Genetic dissection of maize drought tolerance for trait improvement. Mol. Breed..

[3] Dong A., Wang N., Zenda T., Zhai X., Zhong Y., Yang Q., Xing Y., Duan H., Yan X. (2025). ZmDnaJ-ZmNCED6 module positively regulates drought tolerance via modulating stomatal closure in maize. Plant Physiol. Biochem..

[4] Ali S., Tyagi A., Bae H. (2023). ROS interplay between plant growth and stress biology: Challenges and future perspectives. Plant Physiol. Biochem..

[5] Fedoreyeva L.I. (2024). ROS as signaling molecules to initiate the process of plant acclimatization to abiotic stress. Int. J. Mol. Sci..

[6] Li M., Kim C. (2022). Chloroplast ROS and stress signaling. Plant Commun..

[7] Gandin A., Dizengremel P., Jolivet Y. (2021). Integrative role of plant mitochondria facing oxidative stress: The case of ozone. Plant Physiol. Biochem..

[8] Huang L., Liu Y., Wang X., Jiang C., Zhao Y., Lu M., Zhang J. (2022). Peroxisome-mediated reactive oxygen species signals modulate programmed cell death in plants. Int. J. Mol. Sci..

[9] Qi J., Song C.P., Wang B., Zhou J., Kangasjärvi J., Zhu J.K., Gong Z. (2018). Reactive oxygen species signaling and stomatal movement in plant responses to drought stress and pathogen attack. J. Integr. Plant Biol..

[10] Waszczak C., Carmody M., Kangasjärvi J. (2018). Reactive oxygen species in plant signaling. Annu. Rev. Plant Biol..

[11] Leister D. (2019). Piecing the puzzle together: The central role of reactive oxygen species and redox hubs in chloroplast retrograde signaling. Antioxid. Redox Signal.

[12] Huang S., Van Aken O., Schwarzländer M., Belt K., Millar A.H. (2016). The roles of mitochondrial reactive oxygen species in cellular signaling and stress response in plants. Plant Physiol..

[13] Del Río L.A., López-Huertas E. (2016). ROS generation in peroxisomes and its role in cell signaling. Plant Cell Physiol..

[14] Zhang H., Zhu J., Gong Z., Zhu J.K. (2022). Abiotic stress responses in plants. Nat. Rev. Genet..

[15] Miller G., Shulaev V., Mittler R. (2008). Reactive oxygen signaling and abiotic stress. Physiol. Plant.

[16] Sierla M., Waszczak C., Vahisalu T., Kangasjärvi J. (2016). Reactive oxygen species in the regulation of stomatal movements. Plant Physiol..

[17] Noctor G., Mhamdi A., Foyer C.H. (2014). The roles of reactive oxygen metabolism in drought: Not so cut and dried. Plant Physiol..

[18] Qi J., Wang J., Gong Z., Zhou J.M. (2017). Apoplastic ROS signaling in plant immunity. Curr. Opin. Plant Biol..

[19] Hua D., Wang C., He J., Liao H., Duan Y., Zhu Z., Guo Y., Chen Z., Gong Z. (2012). A plasma membrane receptor kinase, GHR1, mediates abscisic acid- and hydrogen peroxide-regulated stomatal movement in Arabidopsis. Plant Cell.

[20] Francoz E., Ranocha P., Nguyen-Kim H., Jamet E., Burlat V., Dunand C. (2015). Roles of cell wall peroxidases in plant development. Phytochemistry.

[21] Shigeto J., Tsutsumi Y. (2016). Diverse functions and reactions of class III peroxidases. New Phytol..

[22] Survila M., Davidsson P.R., Pennanen V., Kariola T., Broberg M., Sipari N., Heino P., Palva E.T. (2016). Peroxidase-generated apoplastic ROS impair cuticle integrity and contribute to damp-elicited defenses. Front. Plant Sci..

[23] Bindschedler L.V., Dewdney J., Blee K.A., Stone J.M., Asai T., Plotnikov J., Denoux C., Hayes T., Gerrish C., Davies D.R. (2006). Peroxidase-dependent apoplastic oxidative burst in Arabidopsis required for pathogen resistance. Plant J..

[24] Daudi A., Cheng Z., O’Brien J.A., Mammarella N., Khan S., Ausubel F.M., Bolwell G.P. (2012). The apoplastic oxidative burst peroxidase in Arabidopsis is a major component of pattern-triggered immunity. Plant Cell.

[25] O’Brien J.A., Daudi A., Finch P., Butt V.S., Whitelegge J.P., Souda P., Ausubel F.M., Bolwell G.P. (2012). A peroxidase-dependent apoplastic oxidative burst in cultured Arabidopsis cells functions in MAMP-elicited defense. Plant Physiol..

[26] López-Castillo L.M., González-Leyzaola A., Diaz-Flores-Rivera M.F., Winkler R., Wielsch N., García-Lara S. (2020). Modulation of aleurone peroxidases in kernels of insect-resistant maize (*Zea mays* L.; Pob84-C3R) after mechanical and insect damage. Front. Plant Sci..

[27] Llorente F., López-Cobollo R.M., Catalá R., Martínez-Zapater J.M., Salinas J. (2002). A novel cold-inducible gene from Arabidopsis, RCI3, encodes a peroxidase that constitutes a component for stress tolerance. Plant J..

[28] Jiao Y., Lv W., Teng W., Li L., Lan H., Bai L., Li Z., Lian Y., Wang Z., Xin Z. (2024). Peroxidase gene TaPrx109-B1 enhances wheat tolerance to water deficit via modulating stomatal density. Plant Cell Environ..

[29] Hu X., Jiang M., Zhang A., Lu J. (2005). Abscisic acid-induced apoplastic H_2_O_2_ accumulation up-regulates the activities of chloroplastic and cytosolic antioxidant enzymes in maize leaves. Planta.

[30] Voothuluru P., Sharp R.E. (2013). Apoplastic hydrogen peroxide in the growth zone of the maize primary root under water stress. I. Increased levels are specific to the apical region of growth maintenance. J. Exp. Bot..

[31] Zhang Q., Liu H., Wu X., Wang W. (2020). Identification of drought tolerant mechanisms in a drought-tolerant maize mutant based on physiological, biochemical and transcriptomic analyses. BMC Plant Biol..

[32] Gentzel I., Giese L., Zhao W., Alonso A.P., Mackey D. (2019). A simple method for measuring apoplast hydration and collecting apoplast contents. Plant Physiol..

[33] Witzel K., Shahzad M., Matros A., Mock H.P., Mühling K.H. (2011). Comparative evaluation of extraction methods for apoplastic proteins from maize leaves. Plant Methods.

[34] Fichman Y., Miller G., Mittler R. (2019). Whole-plant live imaging of reactive oxygen species. Mol. Plant.

[35] Kim D., Langmead B., Salzberg S.L. (2015). HISAT: A fast spliced aligner with low memory requirements. Nat. Methods.

[36] Love M.I., Huber W., Anders S. (2014). Moderated estimation of fold change and dispersion for RNA-seq data with DESeq2. Genome Biol..

[37] Li Y., Niu L., Zhou X., Liu H., Tai F., Wang W. (2023). Modifying the expression of cysteine protease gene PCP affects pollen development, germination and plant drought tolerance in maize. Int. J. Mol. Sci..

[38] Livak K.J., Schmittgen T.D. (2001). Analysis of relative gene expression data using real-time quantitative PCR and the 2(-Delta Delta C(T)) Method. Methods.

[39] Liu H., Song S., Liu M., Mu Y., Li Y., Xuan Y., Niu L., Zhang H., Wang W. (2023). Transcription factor ZmNAC20 improves drought resistance by promoting stomatal closure and activating expression of stress-responsive genes in maize. Int. J. Mol. Sci..

[40] Xu K., Huang X., Wu M., Wang Y., Chang Y., Liu K., Zhang J., Zhang Y., Zhang F., Yi L. (2014). A rapid, highly efficient and economical method of agrobacterium-mediated in planta transient transformation in living onion epidermis. PLoS ONE.

[41] Christensen S.A., Nemchenko A., Park Y.S., Borrego E., Huang P.C., Schmelz E.A., Kunze S., Feussner I., Yalpani N., Meeley R. (2014). The novel monocot-specific 9-lipoxygenase ZmLOX12 is required to mount an effective jasmonate-mediated defense against Fusarium verticillioides in maize. Mol. Plant Microbe Interact..

[42] Zuo W., Chao Q., Zhang N., Ye J., Tan G., Li B., Xing Y., Zhang B., Liu H., Fengler K.A. (2015). A maize wall-associated kinase confers quantitative resistance to head smut. Nat. Genet..

[43] Niu L., Liu L., Wang W. (2020). Digging for stress-responsive cell wall proteins for developing stress-resistant maize. Front. Plant Sci..

[44] Li S., Zheng H., Sui N., Zhang F. (2024). Class III peroxidase: An essential enzyme for enhancing plant physiological and developmental process by maintaining the ROS level: A review. Int. J. Biol. Macromol..

[45] Almagro L., Gómez Ros L.V., Belchi-Navarro S., Bru R., Ros Barceló A., Pedreño M.A. (2009). Class III peroxidases in plant defence reactions. J. Exp. Bot..

[46] Jin T., Sun Y., Zhao R., Shan Z., Gai J., Li Y. (2019). Overexpression of peroxidase gene GsPRX9 confers aalt tolerance in soybean. Int. J. Mol. Sci..

[47] Choi H.W., Kim Y.J., Lee S.C., Hong J.K., Hwang B.K. (2007). Hydrogen peroxide generation by the pepper extracellular peroxidase CaPO_2_ activates local and systemic cell death and defense response to bacterial pathogens. Plant Physiol..

[48] Wally O., Punja Z.K. (2010). Enhanced disease resistance in transgenic carrot (*Daucus carota* L.) plants over-expressing a rice cationic peroxidase. Planta.

[49] Gokce A., Sekmen A.H. (2025). Exploring the regulatory roles of AtGLR3.4 receptors in mitochondrial stress and ROS management in Arabidopsis. Plant Cell Rep..

[50] Kang S.W., Rhee S.G., Chang T.S., Jeong W., Choi M.H. (2005). 2-Cys peroxiredoxin function in intracellular signal transduction: Therapeutic implications. Trends Mol. Med..

[51] Shigeto J., Itoh Y., Hirao S., Ohira K., Fujita K., Tsutsumi Y. (2015). Simultaneously disrupting AtPrx2, AtPrx25 and AtPrx71 alters lignin content and structure in Arabidopsis stem. J. Integr. Plant Biol..

[52] Jin J., Hewezi T., Baum T.J. (2011). Arabidopsis peroxidase AtPRX53 influences cell elongation and susceptibility to Heterodera schachtii. Plant Signal Behav..

[53] Freitas C.D.T., Costa J.H., Germano T.A., de O. Rocha R., Ramos M.V., Bezerra L.P. (2024). Class III plant peroxidases: From classification to physiological functions. Int. J. Biol. Macromol..

[54] Kumar S., Jaggi M., Sinha A.K. (2012). Ectopic overexpression of vacuolar and apoplastic Catharanthus roseus peroxidases confers differential tolerance to salt and dehydration stress in transgenic tobacco. Protoplasma.

[55] Kunieda T., Shimada T., Kondo M., Nishimura M., Nishitani K., Hara-Nishimura I. (2013). Spatiotemporal secretion of PEROXIDASE36 is required for seed coat mucilage extrusion in Arabidopsis. Plant Cell..

[56] Schopfer P., Plachy C., Frahry G. (2001). Release of reactive oxygen intermediates (superoxide radicals, hydrogen peroxide, and hydroxyl radicals) and peroxidase in germinating radish seeds controlled by light, gibberellin, and abscisic acid. Plant Physiol..

[57] Passardi F., Tognolli M., De Meyer M., Penel C., Dunand C. (2006). Two cell wall associated peroxidases from Arabidopsis influence root elongation. Planta.

[58] Duan J., Zhang M., Zhang H., Xiong H., Liu P., Ali J., Li J., Li Z. (2012). OsMIOX, a myo-inositol oxygenase gene, improves drought tolerance through scavenging of reactive oxygen species in rice (*Oryza sativa* L.). Plant Sci..

