# Supplementary material for: Maize Peroxidase ZmPrx25 Modulates Apoplastic ROS Homeostasis and Promotes Seed Germination and Growth Under Osmotic and Drought Stresses
Source: Antioxidants (Basel). 2025 Aug 30;14(9):1067. doi: 10.3390/antiox14091067 (PMC12466370; doi:10.3390/antiox14091067)
Supplement: Supplementary file 1 [file antioxidants-14-01067-s001.zip › antioxidants-3800019-supplementary.pdf]

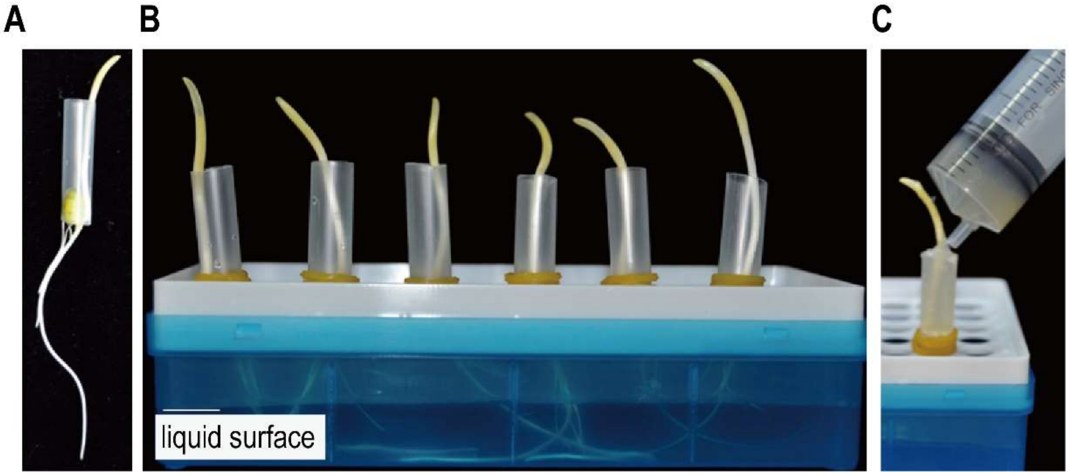

Figure S1. Mesocotyl osmotic stress apparatus. (A) Germinated etiolated seedlings are placed in a plastic straw with an inner diameter of 1 cm, with the mesocotyl about 2 cm long. The length of the straw is adjusted according to the length of the mesocotyl; smaller seeds can be wrapped in sealing film before being placed inside. (B) Assembled device. The root system is normally watered, and below it is a box for 1 ml pipette tips. A rubber ring is added to the lower end of the straw to adjust its height in the insertion hole. (C) A 50 ml syringe is filled with gel (2% agar or 2% agar + 10% PEG 6000).

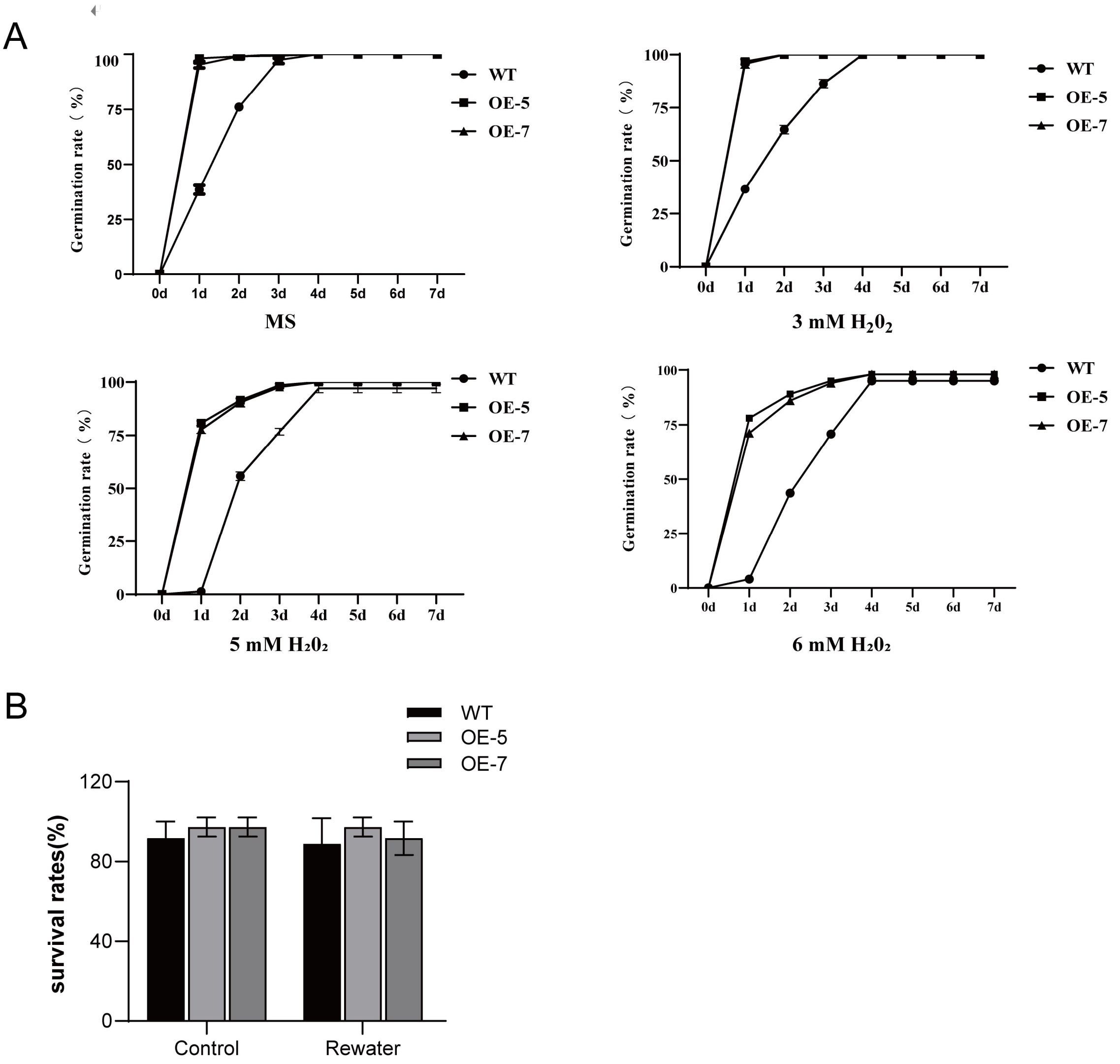

Figure S2. Seed germination rates of Arabidopsis under oxidative stress and survival rates under drought stress. (A) Seed germination rates of Arabidopsis under oxidative stress. WT, wild-type Arabidopsis; OE-5 and OE-7, *ZmPrx25* overexpressed Arabidopsis lines. (B) Survival rates of 15 d seedlings under drought stress. Control, normal growth; Rewater, water withholding for 15 d and 2 d of re-watering.

Table S1. Primers used in this study.

Gene ID	Gene Name	Primer Sequences（5'-3'）	Application
Zm00001d028348	ZmPrx25	F: TTACTGGTGAACTGAAAG<br>R: TATTGGATCTCCTTGACA	RT-qPCR
Zm00001d028348	ZmPrx25	F: ACACGGGGGACTCTTGAC ATGGCGTCCCGTGGC<br>R:AAAGTTCTTCTCCTTTACTGCTG CTTCCGTTGACCATCCTACAGTTCT	Transgenic Arabidopsis/Subcellular localization in Tobacco
Zm00001d028348	ZmPrx25	F: TCTCGAGCTGCAGGCTAGCA ATGGCGTCCCGTGGCAG<br>R: CTCCTCGCCCTTGCTCACG CTTCCGTTGACCATCCTACAGTTCT	Subcellular localization in Onion
Zm00001d028348	ZmPrx25	F: CGACGACGACGACAAGGCA ATGGCGTCCCGTGGCAG<br>R: GCTTGTCGACGGAGCTCGTG CTTCCGTTGACCATCCTACAGTTCT	Prokaryotic protein expression
